# GAINS AND LOSSES OF OLDER ADULTS LIVING IN SUBSIDIZED HOUSING DURING THE COVID-19 PANDEMIC

**DOI:** 10.1093/geroni/igac059.617

**Published:** 2022-12-20

**Authors:** Marcela Blinka, Suzanne Grieb, Katherine Runge, Laura Andes, Carl Latkin, Cynthia Boyd, Thomas Cudjoe

**Affiliations:** JHSOM, Baltimore, Maryland, United States; BEAD CORE, Baltimore, Maryland, United States; Mercy Housing, Denver, Colorado, United States; Mercy Housing, Denver, Colorado, United States; Johns Hopkins University, Baltimore, Maryland, United States; Johns Hopkins University, Baltimore, Maryland, United States; Johns Hopkins School of Medicine, Baltimore, Maryland, United States

## Abstract

Social isolation is prevalent among community dwelling older adults. Low income older adults living in subsidized housing may have increased risk for social isolation. To examine resident experiences and perspectives relating to their social connections during the COVID-19 pandemic, we conducted semi-structured interviews with 13 older adults (62+) who are English, Spanish, and Mandarin speaking recruited from a large non-profit affordable housing organization with communities in 22 states. Twelve housing communities were identified based on distributions of socio-demographic factors and prevalence of self-reported social isolation l in the housing community’s annual survey of residents in order to maximize site diversity. We used qualitative thematic analysis methods to examined participants’ views about their social connections before and during the COVID-19 pandemic, as well as their personal and the housing community’s strategies to mitigate experiences of social isolation. Emerging themes include loss of common facilities and opportunities to socialize with other residents due to COVID-19 restrictions, and increased use of technology to stay connected.

